# Autism and Neurodevelopmental Disorders: The SARS-CoV-2 Pandemic Implications

**DOI:** 10.3390/brainsci13020269

**Published:** 2023-02-06

**Authors:** Antonio Narzisi, Francisco Alcantud-Marin, Yurena Alonso-Esteban, Roberta Battini, Chiara Cantiani, Eugenia Conti, Laura Fusar-Poli, Flavia Lecciso, Annalisa Levante, Luigi Mazzone, Annarita Milone, Valentina Riva, Martina Siracusano, Eugenio Aguglia

**Affiliations:** 1 IRCCS Stella Maris Foundation, 56018 Pisa, Italy; 2 PSiDEHESO Research Team, University of Valencia, 46010 Valencia, Spain; 3 Department of Psychology and Sociology, University of Zaragoza Campus Teruel, 44003 Teruel, Spain; 4 Department of Clinical and Experimental Medicine, University of Pisa, 56126 Pisa, Italy; 5 Child Psychopathology Unit, Scientific Institute, IRCCS Eugenio Medea, Bosisio Parini, 23842 Lecco, Italy; 6 Child Neuropsychiatry Unit, ASL Toscana Centro, 50053 Empoli, Italy; 7 Psychiatry Unit, Department of Clinical and Experimental Medicine, University of Catania, 95123 Catania, Italy; 8 Department of History, Society, and Human Studies, University of Salento, 73100 Lecce, Italy; 9 Child Neurology and Psychiatry Unit, System Medicine Department, Tor Vergata University Hospital of Rome, 00133 Rome, Italy

The Special Issue (SI) “Autism and Neurodevelopmental Disorders: The SARS-CoV-2 Pandemic Implications” is an interesting project that adopted a scientific point of view with important implications in clinical and practical fields. Six contributions were included in this SI between 2020 and 2022: five of them were from Italy [1,2,3,4,5], and one of them was from Spain [6]. Globally, all the articles involved 63 authors and 12 reviewers actively engaged in the field of Autism Spectrum Disorder (ASD) and neurodevelopmental disorders.

The published articles investigated the reaction of children [2,3,4,5], adolescents [2,5] and adults [1] with ASD and neurodevelopmental disorders and their caregivers to the SARS-CoV-2 pandemic.

As Alonso’s interesting review pointed out (2021), the reaction of youth and adults diagnosed with ASD and their parents to the pandemic was not unique. The results of the articles in this SI showed that different reactions are likely attributable to the differences in the severity of ASD, the phenotypic profiles, the age of the participants involved, and the family characteristics.

The study by Fusar-Poli and colleagues [1] from University of Catania (Italy) compared the levels of (i) psychological wellbeing, (ii) family distress, (iii) insomnia, and (iv) resilience experienced by the caregivers of adults with ASD throughout the lockdown period with those experienced by the caregivers of people affected by other neurodevelopmental conditions and psychiatric or relational disorders. They also assessed the factors linked with the levels of individual distress reported by the caregivers over the course of the lockdown.

A web survey disseminated via social media was completed by 383 individuals, of which 141 were primary caregivers of people with ASD, and 242 of them were primary caregivers of people with other conditions. The results did not show any significant difference in the considered psychological variables between the caregivers of ASD and non-ASD people. Lower resilience and younger ASD family members’ age were significantly associated with higher distress in the caregivers of ASD subjects. The results do not validate the hypothesis that caregivers of ASD subjects experienced more distress than other caregivers over the course of the lockdown.

The objective of the Siracusano and colleagues’ study [2] conducted at Tor Vergata University of Rome (Italy) was to compare the levels of parental stress during the COVID-19 pandemic between children with four different conditions: (1) ASD, (2) attention deficit and hyperactivity disorder (ADHD), (3) Rett syndrome (RTT), and (4) Sotos syndrome (SS), and those with typical development (TD).

Two hundred and twenty-two Italian parents of youths from 2 to 19 years old in four disability groups (ASD, ADHD, RTT, and SoS) and one control group (TD) were included in a standardized assessment of stress using the Parental Stress Index Short Form [3]. The main results showed greater levels of stress among the parents of children with a disorder than among those with children who developed typically. The main conclusion of Siracusano’s study consists of the fact that parenting a child with a developmental disorder during the COVID-19 outbreak led to significantly more stressed caregivers, which is therefore related to the management difficulties of child’s disability. Furthermore, this study highlights the need to support not only individuals with special needs but also their own caregivers.

The study by Levante and colleagues [3] at the University of Salento (Italy) aimed to explore the psychological impact of the pandemic on families with children with ASD. To be specific, the study aimed at testing the interplay between (i) parental distress, (ii) the children’s positive and (iii) negative emotional responses, and (iv) their adaptive behaviors in terms of their ability to play independently during the lockdown. Furthermore, the authors tested the same mediation models comparing families with children with ASD and typically developing (TD) ones. In this study, 120 parents of children between 5 and 10 years old (53 with ASD) were recruited through popular social media platforms (i.e., WhatsApp and Facebook) and via a snowball sampling technique.

In the four tested mediation models, the children’s positive and negative emotional responses mediated the impact of parental distress on the children’s ability to play independently. In comparing the ASD and TD groups, the parents of children with ASD reported that their children showed more positive emotions, but less ability to play than the parents of TD children did. Additionally, families with an ASD child have expressed that there were greater behavioral issues (i.e., stereotypes and repetitive behaviors) during the lockdown and more parental distress. These results inform the intervention programs designed for parents to diminish distress and to develop coping procedures to better manage the caregiver–child relationship.

The study of Cantiani and colleagues [4] at the Scientific Institute, IRCCS Eugenio Medea of Bosisio Parini (Lecco, Italy), investigated the emotional and behavioral development of preschoolers with typical development (TD) and preschoolers at high risk for developing neurodevelopmental disorders (HR-NDD).

The study involved 90 preschoolers. Before the lockdown (T0), they were clinically evaluated with the checklist Child Behavior Check List 1.5–5 (CBCL/1.5–5) [7]. They were evaluated again during the emergency (T1) using CBCL/1.5–5 and a questionnaire investigating the environmental factors characterizing the specific period. Modifications to the CBCL/1.5–5 profiles between T0 and T1 were assessed. Globally, without regard to the familial risk, the scores on the CBCL/1.5–5 scales at T1 were higher than they were at T0. Associations emerged between the delta scores, reflecting worse scores on specific CBCL scales and for clinical and environmental factors. These findings established the negative impact of the lockdown on the children’s emotional/behavioral profiles and focus attention on the need for strategic approaches in the preschooler age range, especially for more vulnerable children, owing to environmental factors and pre-existing emotional problems.

The study by Cantiani and colleagues provides empirical findings on the effects of the SARS-CoV-2 lockdown on preschool children with and without familial risk for NDD. The findings of this study showed the negative impact of SARS CoV-2 restrictions on the emotional and behavioral profiles of preschoolers, regardless of NDD risk, but there was a worse effect on the children with previous emotional and behavioral vulnerabilities.

Conti and colleagues [5] at the Scientific Institute IRCCS Stella Maris of Calambrone (Pisa, Italy) carried out a study to examine lockdown-related emotional and behavioral modifications in the pediatric neuropsychiatric population.

Families with children aged from 1.5 to 18 years were contacted to fill out two online questionnaires: (1) a questionnaire to assess the impact of the SARS-CoV-2 emergency on families with youths with neuropsychiatric disorders, and (2) CBCL 1.5–5 or 6–18 [7,8] to (a) compare the scores during the lockdown with previous ones and (b) investigate the influence of variables such as age, diagnosis grouping, and financial difficulties. One hundred and forty-one parents filled out the questionnaires via the REDCap online platform. The findings show that anxiety and somatic problems worsened in preschool children. In contrast, obsessive-compulsive, post-traumatic, and thought problems worsened in older children (6–18 years). The younger children had “protective” symptoms, while the financial difficulties experienced by families during the lockdown were the cause of the psychiatric symptoms in the 6–18 years subpopulation.

In the article of Alonso-Esteban and colleagues [6] at the University of Valencia, a systematic search was conducted based on the studies that have been published since the beginning of the COVID-19 pandemic regarding the consequences of the restraint measures on ASD subjects and their caregivers.

Six academic research databases were examined, and ten studies were selected. The studies were grouped based on their theoretical focus, methodology, and target population. The findings showed a rise in stress and a reduction in psychological well-being among the subjects with ASD. Furthermore, in the selected studies, the findings were discrepant because of certain variables such as age, ASD severity, or the type of family features.

To conclude, we want to thank all of the authors who submitted their work to this Special Issue and the reviewers for dedicating their time to improving the quality of the published manuscripts.
